# What impact did the creation of Local Health Care Co-operatives have on indicators of practice resources and activity?

**DOI:** 10.1186/1472-6963-8-104

**Published:** 2008-05-16

**Authors:** Gary McLean, Matt Sutton

**Affiliations:** 1 General Practice & Primary Care, University of Glasgow, 1 Horselethill Road Glasgow, G12 9LX, UK; 2 Health Economics Research Unit, University of Aberdeen, Polworth Building, Fosterhill, Aberdeen, AB25 2ZD, UK

## Abstract

**Background:**

The creation of Local Health Care Cooperatives (LHCCs) in Scotland in 1999 was typical of attempts to encourage voluntary integration and co-operation between health care providers. One of the three stated objectives of their introduction was to tackle inequalities and improve access to care.

**Methods:**

We used administrative data on all general practices in 1999 and 2003 to examine whether LHCCs had any measurable impact on six indicators of practice resources and activity. We compare three groups (participant, non-participant, and ineligible practices) through regression analysis of changes over time in group means and within-group inequality (measured using Gini coefficients). In addition, for participants we measure changes in the variation between and within LHCCs.

**Results:**

Despite having similar registered populations to participants, non-participants had lower levels of resources at the start of the period and this differential widened over time. The changes over time in the activity indicators were similar across the three groups. There was little evidence that inequality between LHCC practices narrowed more than in the other two groups. Practices within LHCCs appear to be become more homogenous while variation increased between LHCCs.

**Conclusion:**

The mixed messages from our examination of resources and activity indicators demonstrates that there are likely to be important lessons to be learned from the brief experiment with LHCCs. Clear objectives that are evaluated using a battery of simple performance indicators may help to ensure demonstrable change in future initiatives to foster integration and co-operation.

## Background

The formation of Local Health Care Cooperatives (LHCCs) in Scotland in April 1999 emerged out of the White Paper 'Designed to Care' [1] and represented a major change in the way that primary health care services were to be organised. The creation of LHCCs reflected the continuing importance of a shift towards a primary care led NHS as a health policy goal in the UK [2] and was part of wider attempts to develop integrated and co-operative primary health care organisations, which revolve around primary care [3,4]. They were a classic example of the philosophy in Scotland following devolution in the UK that health care improvement could be achieved by greater collaboration and co-operation between integrated health care organisations [5]

The Minister for Health and Community Care identified three priority areas for action by LHCCs [6]:

• to increase patient and public involvement;

• to increase the scale of partnership working with social work, the acute sector and voluntary agencies; and

• to tackle inequalities and improve access to primary care services.

LHCCs were accountable to Primary Care Trusts (PCTs) and comprised voluntary groups of GPs and primary healthcare professionals. Although LHCCs were an operational unit within the PCT, they acted as separate management entities. By 2003 there were 79 LHCCs across Scotland.

The organisational structures implemented through the introduction of LHCCs were substantively different from those adopted in England and Wales [7]. The approach adopted in Scotland favoured a more collaborative style with the general guidance for LHCCs stressing that there was to be no single model and that the exact scope and functions of the LHCC would be determined by discussion and agreement between member practices and the PCT. Unlike Primary Care Groups (PCGs) in England, LHCCs did not have the power to commission secondary care services for their local population. They were also to have less budgetary control than PCGs for, while they were responsible for managing and operating their budget, the cash was to be administered by the PCT, to whom LHCCs were financially accountable.

The role of LHCCs ended in April 2004 when they, along with Acute Hospital Trusts and Primary Care Trusts, were replaced by Community Health Partnerships (CHPs). The CHPs were intended to evolve from LHCCs and it is therefore important to assess the impact and possible future consequences of the experiment with LHCCs. In particular, the process of change initiated by the introduction of LHCCs may mean that much professional time and effort may have been expended on trying to understand new roles and structures instead of developing patient care [7].

Previous studies have focused on factors concerning the development [7,8] and management of LHCCs [9,10] or sought to elicit stakeholder opinion on their preferred form of LHCCs [11]. Within this body of work it has been argued that LHCCs represent a shift back to the 'meso-level' in service planning and purchasing. As such there is a fear that perceived micro-level benefits of fund holding will be lost, while uncertainties remain regarding the capacity of LHCCs to incorporate GPs into a collaborative approach to strategic decision making [3].

Research undertaken by the LHCC Best Practice Group found that attempts to engage patients were limited and needed to be stepped up to increase responsiveness and accountability to local health care users [12]. Furthermore in order to facilitate co-operation among participating practices, LHCCs needed to focus on issues of leadership, organisation and involvement in decision-making [13]. Research has indicated that some LHCC boards may have been too large to work effectively and that size did not have a significant effect on clinical governance activities or public and patient involvement [13].

A survey undertaken of Scottish practices suggested that four types of factors affected a general practice's decision to become a member of an LHCC [14]. These factors were: access to NHS and local authority facilities; GP workload; population morbidity and deprivation; and history of integration of primary care. Furthermore evidence showed that a concern over resources from general practices increased the probability of LHCC membership, whereas rurality decreased it [15]. Work that has focused on integration has found two distinct groups of LHCCs, with one achieving a higher extent of integration than the other. Those LHCCs that were less integrated could not be distinguished on the basis of area, population or GP characteristics. The extent to which a general practice integrated within an LHCC depended on the LHCC that the practice had joined [14].

Much of the existing research has focused on the processes by which LHCCs integrated. There has been no research on whether the creation of LHCCs had an impact on quantitative indicators of the resources available to practices and on their levels of activity. In this paper, we examine whether LHCCs affected mean levels of practice resources and activity by comparing data measured at the time that LHCCs were introduced in 1999 and just before they were abolished in 2003. We also examine whether LHCCs influenced the amount of variation between practices over this same period. We propose that this provides some evidence on how successful LHCCs were in addressing one of their key priorities; to tackle inequalities and improve access to primary care services.

Previous work that has examined LHCC integration has either focused on entire LHCCs or been restricted to a comparison with those practices that have chosen not to form LHCCs. This paper provides a fuller picture with regard to the integration of LHCCs by, for the first time, comparing all general practices in Scotland. We compare three groups of practices: (i) LHCC participants; (ii) non-participants; and (iii) practices in areas that chose not to form LHCCs, which we label 'ineligible practices'.

## Methods

Practices were able to join and/or leave LHCCs throughout the period 1999–2003. Complete information on LHCC membership was not obtained centrally until March 2003. We have therefore been restricted to assigning LHCC membership for the entire period based on the snapshot as at March 2003. Backmapping was undertaken of practices that opened or closed throughout the period and practices that were closed in March 2003 were assigned to the LHCCs of their forebears. The Health Board areas that chose not to form LHCCs were Orkney, Shetland and the Western Isles, as well as mainland West Lothian.

We draw on a number of data sets for our indicators. These were obtained from a central NHS information agency (the Information and Statistics Division) at practice level, on the agreement that no data would be presented for individual practices and no further ethical approval was required. We compare practice-level figures in 1999 and 2003. Population data were obtained from the Community Health Index (CHI) database. All indicators were expressed per head of registered population unless otherwise indicated.

We obtained three indicators of the resources available at each practice. The first is the number of whole-time equivalent GPs per 1,000 registered patients. Data on the number of whole-time equivalent GPs by practice as at October 1998 and October 2002 were obtained from the General Medical Practitioner Database. The second indicator is the annual payment made to practices to partially reimburse them for the costs of employing staff. It reflects the size of the primary health care team (excluding GPs). The third indicator reflects all General Medical Services (GMS) payments made to the practice, excluding payments made for staff, premises and IT. Since these data are not available for Personal Medical Services (PMS) practices, these practices are excluded from analysis of this variable.

We use two indicators of practice prescribing behaviour, both derived from the Prescribing Information System. The volumes of statin drugs (British National Formulary (BNF) 2.12) and anti-depressants (BNF 4.3.1–4.3.4) dispensed in the community were converted to Defined Daily Doses. Data on the numbers of referrals made to outpatient specialties of Scottish hospitals were obtained from Scottish Morbidity Records (SMR00).

Figures on deprivation are derived from a modified version of the Scottish Index of Multiple Deprivation (mSIMD) [14]. We measure the extent of rurality using information on the proportion of patients for which the practice claims road mileage payments. These were payments made to practices if individual patients lived more than a stated distance away from the practice and were intended to compensate practices for the additional costs of serving dispersed populations.

We examine three main features of the indicators: (i) cross-sectional differences and changes over time in group means; (ii) cross-sectional differences and changes over time in the extent of inequality within groups; and (iii) changes over time in the variation within and between LHCCs.

We analyse changes in both the mean and the extent of variation in each indicator to reflect the dual role of LHCCs. We anticipate that efforts to improve access to services will lead to changes in the mean levels of practice resources and healthcare activity. At the same time, LHCCs had a significant clinical governance role and were concerned with addressing the substantial inequalities in resourcing and access between practices in the same neighbourhood. Thus, we expect LHCCs to reduce variations between practices. We calculate this extent of variation, or 'inequality', within groups using Gini coefficients. The Gini coefficient is widely used to measure income inequality and has been suggested for measuring health inequality by researchers at the World Health Organisation [16,17].

The Gini coefficient for the participating practices indicates whether the level of variation between all participating practices has changed over time. But changes in the extent of variation may reflect two phenomena (i) changes in the extent of variation *between *LHCCs and/or (ii) changes in the extent of variation *within *LHCCs. It is important to distinguish between these two possible effects because we would anticipate that LHCCs may have brought about more homogeneity within LHCCs but the effect on between-LHCC variation is unknown.

### Analysis of group means

We begin by comparing levels of, and change over time in, mean values of the various indicators across the three groups. We stacked the data for the first and second periods and used regression analysis to calculate the (population-weighted) mean values and standard errors, and test for significant differences. We used robust standard errors, which allows the variance to differ between groups and time periods, and weighted the analysis by population size.

Formally, the regression model can be written as:

$$y_{ijt}=\beta_{0}+\beta_{1}t+\beta_{2}D_{ijt}^{1}+\beta_{3}D_{ijt}^{1}t+\beta_{4}D_{ijt}^{2}+\beta_{5}D_{ijt}^{2}t+\varepsilon_{ijt},\;\varepsilon_{ijt}\sim N(0,\sigma_{jt}^{2})$$

in which *y*_*ijt *_is the value of the indicator for practice *i *in group *j *at time *t*, *t *is a binary variable taking a value of zero in the first year (1999) and one in the final year (2003), *D*^1 ^and *D*^2 ^are binary variables indicating that the practice is a non-participating practice or an ineligible practice respectively, and the *β*-parameters are to be estimated. This structure allows us to test a range of hypotheses about the differences in the group means (see Table 1).

**Table 1:** Hypotheses tested based on estimated regression parameters

**Hypothesis**	**Test **
No significant change over time in the average for participants	*β*_1_= 0
Non-participants are not significantly different from participants at baseline	*β*_2 _= 0
No significant change over time in the average for non-participants	*β*_3 _- *β*_2 _= 0
Non-participants are not significantly different from participants in second period	*β*_3 _- *β*_1 _= 0
Ineligibles are not significantly different from participants at baseline	*β*_4 _= 0
No significant change over time in the average for ineligibles	*β*_5 _- *β*_4 _= 0
Ineligibles are not significantly different from participants in second period	*β*_5 _- *β*_1 _= 0

### Extent of inequality within groups

The Gini coefficient can be estimated using a convenient regression approach [17], which has the advantage of providing standard errors when using the Newey-West correction. In this framework, we can adopt the same regression model as for comparing group means.

Formally, this regression model can be written as:

$$\begin{array}{l} 2\sigma_{rjt}^{2}\frac{y_{ijt}}{{\bar{y}}_{jt}}=\alpha_{0}+\alpha_{1}t+\alpha_{2}D_{ijt}^{1}+\alpha_{3}D_{ijt}^{1}t+\alpha_{4}D_{ijt}^{2}+\alpha_{5}D_{ijt}^{2}t+\\ \;\;\;\;\;\delta_{0}R_{ijt}+\delta_{1}R_{ijt}t+\delta_{2}D_{ijt}^{1}R_{ijt}+\delta_{3}D_{ijt}^{1}R_{ijt}t+\delta_{4}D_{ijt}^{2}R_{ijt}+\delta_{5}D_{ijt}^{2}R_{ijt}t+u_{ijt} \end{array}$$

in which *R*_*ijt *_represents the fractional relative rank of practice *i *within group *j *at time *t*, *σ*^2^_*rjt *_is the variance of the fractional ranks within group *j *at time *t*, *u*_*ijt *_are error-terms with zero mean and a lag structure of order 1 and the other variables are defined as before. The *δ*-parameters provide estimates of the Gini coefficients for each group. We can test whether the extent of inequality varies between groups and over time using the same battery of tests as are applied to the group means (the *β*-parameters) listed in Table 1.

### Variation within and between LHCCs

We summarise the total variation between participating practices using the coefficient of variation (*CoV*). We use regression analysis to distinguish between the between-LHCC and within-LHCC variation. Restricting the analysis to participating practices only, we estimate a regression model for each of the indicators including a series of dummy variables representing membership of a particular LHCC. From this, we can calculate the proportion of variation explained by LHCC membership (the *R*^2^-statistic). The extent of between-LHCC variation is given by *CoV*_*B *_= CoV*R^2 ^and the extent of within-LHCC variation is given by *CoV*_*W *_= CoV*(1-*R*^2^). An increase in the value of *CoV*_*B *_over time indicates that there is more variation between LHCCs. An increase in *CoV*_*W *_indicates that practices within the same LHCC have become less alike.

## Results

Table 2 shows the numbers of practices and the sizes of the population registered in each of the three groups of practices in 1999 and 2003. The participant group is the largest by far and it should be noted, therefore, that we are more likely to find that changes over time for this group are significant at any given level of significance.

**Table 2:** Characteristics of populations registered with three groups of practices

**Indicator**	**Participants**	**Non-participants**	**Ineligible**
Number of practices	961	24	64
Registered population ('000s)	4,987	117	244

*Age composition (proportions)*			
0 – 14 years	0.169	0.172	0.185
15 – 45 years	0.447	0.448	0.439
46 – 60 years	0.186	0.192	0.204↑
61 – 74 years	0.126	0.124	0.107↑
75 years plus	0.072	0.064	0.063↑

Proportion rural patients	0.085	0.086	0.154↑
Mean deprivation score*	0.023	0.017	-0.275

↑ Indicates statistically significant difference(p < 0.05) from participants.

* Deprivation is measured using the modified Scottish Index of Multiple Deprivation. It is a z-score with mean zero. Positive values indicate above average deprivation.

The age compositions of the populations registered with participant and non-participant practices are very similar. The populations registered with ineligible practices are slightly younger than those registered with LHCC participants. Table 2 also shows the deprivation and rural characteristics for the three groups. The mean mSIMD values for the three groups indicate that ineligible practices have the lowest level of deprivation on average. Ineligible practices also had a higher proportion of rural patients, while participant and non-participant practices had similar proportions of rural patients.

### Group Means

Table 3 shows that ineligible practices had significantly greater numbers of GPs and levels of total resources than the other two groups in 1999. All three groups increased their resources in terms of numbers of GPs, with non-participants showing the greatest increase. However, because of the sample size, the changes over time were only statistically significant for participants. In terms of staff costs all three groups recorded a significant increase. Non-participants experienced the smallest increase (20%, compared to 56% for ineligible practices and 40% for participants). By 2003 ineligible practices had significantly more staff resources than participants. For total resources all three groups experienced increases over time of around 20%.

**Table 3:** Group means by year and group

	**1999**			**2003**		
**Indicator**	**Participants**	**Non-participants**	**Ineligible**	**Participants**	**Non-participants**	**Ineligible**
*Resources*						
GPs (WTE per 1,000 patients)	0.649	0.634	0.727↑	0.654*	0.652	0.736↑
Staff costs (£ per patient)	10.91	9.20	11.03	15.30*	11.59*	17.21* ↑
Total resources (£ per patient)	41.50	40.63	47.01↑	50.01*	48.57	56.21* ↑

*Prescribing (DDDs per patient)*						
Statins	5.97	5.23	4.91↑	22.81*	22.93*	22.31*
Anti-depressants	13.51	12.72	13.22	20.83*	20.51*	20.13*
*Access to secondary care*						
Referrals per patient	200.7	201.2	187.8↑	187.1*	183.1	180.1↑

* Indicates statistically significant change over time (p < 0.05).

↑ Indicates statistically significant difference from participants in time period (p < 0.05).

In 1999, non-participant and ineligible practices had lower statin and anti-depressant prescribing than participants, though the difference was statistically significant only for statin prescribing by ineligible practices. All three groups experienced significant increases over time and there were no significant differences between the groups by 2003. In 1999, ineligible practices had significantly lower referral rates. All three groups experienced reductions in referral rates over time, though the change was only significant for participants. By 2003, the difference between ineligible and participant practices had narrowed but remained significant. Non-participants then had lower referral rates than participants but the difference remained insignificant.

### Variation within Groups

Table 4 indicates that there was significantly more inequality in numbers of GPs between non-participants and between ineligible practices in 1999 than between participants. The degree of inequality between participants increased slightly while it fell within the other two groups. In 2003, inequality remained significantly higher amongst non-participants.

**Table 4:** Gini coefficients by year and group

	**1999**			**2003**		
**Indicator**	**Participants**	**Non-participants**	**Ineligible**	**Participants**	**Non-participants**	**Ineligible**
*Resources*						
GPs (WTE/1,000 patients)	0.113	0.214↑	0.173↑	0.118*	0.185↑	0.168
Staff costs (£ per patient)	0.258	0.317	0.496↑	0.178*	0.383↑	0.264* ↑
Total resources (£ per patient)	0.078	0.128↑	0.184	0.083*	0.122↑	0.186

*Prescribing (DDDs per patient)*						
Statins	0.212	0.231	0.170↑	0.182*	0.160	0.162↑
Anti-depressants	0.151	0.200	0.116↑	0.150*	0.176	0.120↑

*Access to secondary care*						
Referrals per patient	0.118	0.132	0.096↑	0.132*	0.127	0.106↑

* Indicates statistically significant change over time.

↑ Indicates statistically significant difference from participants in time period.

For staff costs, participants began the period with the lowest levels of inequality. The levels of inequality fell significantly over time between participants and between ineligible practices, while the level of inequality increased between non-participants. At the end of the period there was significantly less inequality in staff costs between participants than within the other two groups.

In 1999, the inequality in total resources was lowest between participant practices. Although this increased significantly over time, this group still finished the period with the lowest level of inequality of all the groups.

With regard to statin and anti-depressant prescribing and referrals, variation amongst ineligible practices was significantly lower than amongst participants. Variation amongst non-participants was higher than amongst participants. The degree of variation in both prescribing indicators fell in all three groups and fell to the greatest extent for non-participants. The degree of variation in referral rates rose significantly over time for participants so that this group ended the period with the greatest extent of variation between practices.

### Within and between LHCC variation

Amongst the participants, greater levels of overall variation were found in 1999 than in 2003 with the exception of staff costs and statin prescribing (Table 5).

**Table 5:** Coefficients of variation within and between LHCCs

	**Total**		**Between LHCCs**		**Within LHCCs**	
**Indicator**	**1999**	**2003**	**1999**	**2003**	**1999**	**2003**
*Resources*						
GPs (WTE/1,000 patients)	0.076	0.095	0.024	0.026	0.052	0.069
Staff costs (£ per patient)	0.221	0.128	0.108	0.053	0.113	0.075
Total resources (£ per patient)	0.075	0.083	0.026	0.035	0.049	0.048

*Prescribing (DDDs per patient)*						
Statins	0.158	0.116	0.047	0.040	0.111	0.076
Anti-depressants	0.073	0.074	0.021	0.024	0.052	0.050

*Access to secondary care*						
Referrals per patient	0.047	0.059	0.015	0.024	0.032	0.035

The increase in overall variation in numbers of GPs of 25% was explained by an 8% increase in the variation between LHCCs and a 32% rise in the variation between practices within the same LHCC. Staff costs saw a fall in the overall level of variation by 43%. This was made up of a 51% fall between LHCCs and a 35% fall for practices within LHCCs. In contrast, total resources saw an overall increase in variation of 11%. This was due to a rise in variation between LHCCs of 31% compared to a small fall within LHCCs of 2%.

A mixed picture with regard to prescribing and treatment procedures and levels of variation also emerged. Figures for statin prescribing indicate falls in overall (27%), between LHCC (15%) and within LHCC (32%) variation. Anti-depressant prescribing saw a minimal overall increase in variation with variation between LHCCs increasing by 13% and variation within LHCCs falling by 5%. For referrals an overall rise in variation (25%) was matched by increases in variation between LHCCs (35%) and within LHCCs (6%).

## Discussion

Our analysis shows mixed evidence of the impact of LHCCs across four dimensions. First we have examined whether LHCCs facilitated access to more resources for their member practices. Despite finding little difference in the population characteristics of participant and non-participant practices, we found that non-participants had lower levels of resourcing prior to the introduction of LHCCs and had fallen further behind in terms of reimbursed staff costs by 2003. However, the participants experienced similar changes to the ineligible practices. This suggests that the widening difference between participants and non-participants was not due to the positive benefits of participation but rather the negative consequences of non-participation. Given that previous research has shown that general practices located in deprived areas and covering populations with high levels of morbidity exhibited a high probability of joining an LHCC [14], the fact that participants have increased their share of resources over non-participants would appear notable.

Second, we have found that, regardless of the resourcing changes, all three groups showed similar mean changes in the activity indicators. Increases in the level of statin prescribing, in particular, reflect a similar trend found in England and Wales [19]. We have not attempted to model formally the relationships between changes in levels of input and levels of output and this would be an important priority for further research, but our findings suggest that non-participation in LHCCs did not lead to deleterious changes in output for non-participating practices.

Third, previous research has shown that there is scope to improve the consistency of prescribing, and this is an area where it is believed LHCCs could have a major impact [9]. Indeed it was found that work was underway or had been completed on promoting the exchange of prescribing data and the development of a prescribing policy in most LHCCs [9]. However, although there is some evidence of a reduction in the variation in statin prescribing between participant practices, similar falls have been recorded in the other two groups. Indeed in terms of both statin and anti-depressant prescribing non-participants recorded a larger fall in variation. This may indicate that the suggested increase in homogeneity between participating practices may not be due entirely to LHCC membership but rather have emerged from a more general trend in general practices on the Scottish mainland.

Finally, we examined whether changes in the variation between practices participating in LHCCs reflected more homogeneity within LHCCs and/or more homogeneity between LHCCs. On four of the six indicators we found that between-LHCC variation increased over time. Within-LHCC variation increased on only two of the six indicators. There is therefore some evidence that practices within LHCCs became more alike while LHCCs as a whole became more dissimilar. This may seem a natural consequence of local integration but was inconsistent with the central government's desire to have common prescribing and referral policies across the country. The increase in between-LHCC variation may also have important effects as LHCCs merge into a smaller number of CHPs with greater influence, governance and responsibility. It also contains important messages for other structural organisational changes for other countries such as the recent realignment of PCTs in England.

## Conclusion

These mixed messages from our examination of resourcing and activity indicators demonstrate that there are likely to be important lessons to be learned from the brief experiment with LHCCs. Clear objectives that will be evaluated using a battery of simple performance indicators may help to ensure demonstrable change. These should assess average levels of achievement and the successfulness of attempts to move to a more equitable distribution of resources and equalise standards of care across and within CHPs.

## Competing interests

The authors declare that they have no competing interests.

## Authors' contributions

GMcL and MS conceptualised the study, conducted the data analysis, wrote and revised the manuscript. GM is the guarantor.

## Pre-publication history

The pre-publication history for this paper can be accessed here:



## References

[B1] The Scottish Office (1997). 'Designed to Care': Renewing the NHS in Scotland.

[B2] Craig N, McGregor S, Drummond N, Fischbacher M, Iliffe S (2002). Factors affecting a shift towards a 'primary care-led' NHS: a qualitative study. British Journal of General Practice.

[B3] Simeons S, Scott A The determinants of integrated primary health care organisations: an empirical study of local healthcare co-operatives. HERU Discussion paper 2002; 02/02.

[B4] Goddard M, Mannion R (1998). From competition to co-operation: new economic relationships in the National Health Service. Health Econ.

[B5] Alvarez-Rosete A, Bevan G, Mays N, Dixon J (2005). Effect of diverging policy across the NHS. BMJ.

[B6] The Scottish Office (2000). Our National Health: A plan for action; a plan for change.

[B7] Hopton J, Heaney D (1999). The development of local health care co-operatives in Scotland. BMJ.

[B8] Drummond N, Iliffe S, McGregor S, Craig N, Fischbacher M (2001). Can primary care be both patient centred and community led?. Journal of Management in Medicine.

[B9] Audit Scotland (2001). Paying dividends- Local Healthcare Co-operatives Bulletin.

[B10] Simeons S, Scott A (2003). How are Scottish integrated primary care organisations managed?. Journal of Health Organisation and Management.

[B11] Royal College of General Practitioners and British Medical Association (2000). LHCC's-The first year and the way ahead. LHCC Conference report.

[B12] LHCC Best Practice Group (2001). Connecting Communities with the NHS: Final Report.

[B13] Simeons S, Scott A Why do general practices integrate? A quantitative analysis of the factors affecting integration in primary care. HERU Discussion paper; 2001; 01/01.

[B14] Simeons S, Scott A Working together in primary care: measuring the extent of integration with local health care co-operatives. HERU Discussion paper 2001; 04/01.

[B15] McConnachie A, Sutton M, Watt G (2003). The Characterisation of Deprivation for General Practices in Glasgow: Final report for the Greater Glasgow NHS Primary Care Trust.

[B16] Gakidou E, Murray C, Frenk J (2000). Defining and measuring health inequality. Bulletin of the World Health Organisation.

[B17] Van Doorslaer E, Jones AM (2003). Inequalities in self-reported health: validation of a new approach to measurement. Journal of Health Economics.

[B18] Kakwani NC, Wagstaff A, van Doorslaer E (1997). Socio-economic inequalities in health: measurement, computation and statistical inference. Journal of Econometrics.

[B19] DeWilde S, Carey IM, Bremner SA, Richards N, Hilton SR, Cook DG (2003). Evaluation of Statin prescribing 1994–2001: a case of ageism but not of sexism. Heart.

